# Anti-GD2 Immunoliposomes for Targeted Delivery of the Survivin Inhibitor Sepantronium Bromide (YM155) to Neuroblastoma Tumor Cells

**DOI:** 10.1007/s11095-018-2373-x

**Published:** 2018-03-07

**Authors:** Shima Gholizadeh, Emmy M. Dolman, Rebecca Wieriks, Rolf W. Sparidans, Wim E. Hennink, Robbert J. Kok

**Affiliations:** 1 Department of Pharmaceutics, Utrecht Institute for Pharmaceutical Sciences, Utrecht University, Utrecht, the Netherlands; 2 Department of Oncogenomics, Academic Medical Center, University of Amsterdam, Amsterdam, the Netherlands; 3 Princess Maxima Center for Pediatric Oncology, Utrecht, the Netherlands; 4 Department of Chemical Biology and Drug Discovery, Utrecht Institute for Pharmaceutical Sciences, Utrecht University, Utrecht, the Netherlands

**Keywords:** immunoliposomes, neuroblastoma cells, sepantronium bromide (YM155), targeted delivery

## Abstract

**Purpose:**

Sepantronium bromide (YM155) is a hydrophilic quaternary compound that cannot be administered orally due to its low oral bioavailability; it is furthermore rapidly eliminated via the kidneys. The current study aims at improving the pharmacokinetic profile of YM155 by its formulation in immunoliposomes that can achieve its enhanced delivery into tumor tissue and facilitate uptake in neuroblastoma cancer cells.

**Methods:**

PEGylated YM155 loaded liposomes composed of DPPC, cholesterol and DSPE-PEG_2000_ were prepared via passive film-hydration and extrusion method. Targeted (i.e. immuno-)liposomes were prepared by surface functionalization with SATA modified monoclonal anti-disialoganglioside (GD2) antibodies. Liposomes were characterized based on their size, charge, antibody coupling and YM155 encapsulation efficiency, and stability. Flow cytometry analysis and confocal microscopy were performed on IMR32 and KCNR neuroblastoma cell lines. The efficacy of developed formulations were assessed by in-vitro toxicity assays. A pilot pharmacokinetic analysis was performed to assess plasma circulation and tumor accumulation profiles of the developed liposomal formulations.

**Results:**

YM155 loaded immunoliposomes had a size of 170 nm and zeta potential of −10 mV, with an antibody coupling efficiency of 60% andYM155 encapsulation efficiency of14%. Targeted and control liposomal formulations were found to have similar YM155 release rates in a release medium containing 50% serum. An in-vitro toxicity study on KCNR cells showed less toxicity for immunoliposomes as compared to free YM155. In-vivo pharmacokinetic evaluation of YM155 liposomes showed prolonged blood circulation and significantly increased half-lives of liposomal YM155 in tumor tissue, as compared to a bolus injection of free YM155.

**Conclusions:**

YM155 loaded immunoliposomes were successfully formulated and characterized, and initial in-vivo results show their potential for improving the circulation time and tumor accumulation of YM155.

**Electronic supplementary material:**

The online version of this article (10.1007/s11095-018-2373-x) contains supplementary material, which is available to authorized users.

## Introduction

Neuroblastoma (NB) is an aggressive malignancy of the sympathetic nervous system and is the most frequently occurring type of solid extracranial tumor in children (1). Although the survival rate in low-risk patients is currently over 90%, children with high-risk neuroblastoma currently have a very poor prognosis. For these patients, the 5-year disease free survival rate is between 25%–35%, despite many patients undergoing aggressive multi-modality therapies (i.e. *combinations of chemotherapy*, surgery, stem cell rescue and *radiation therapy)* (2). This highlights the urgent need for new therapeutic strategies (1,3,4).

The pathology of neuroblastoma (as well as most other types of cancer) is complex and differs between individual patients. Anti-apoptotic proteins have been shown to play a role in tumor development, thus making such proteins promising drug targets. One of these proteins is survivin, which is encoded by the ‘baculoviral inhibitor of apoptosis repeat-containing 5’ (BIRC5) gene. Over-expression of the survivin-encoding gene has been shown to correlate strongly to a poor patient prognosis, metastatic spread and (increased) resistance to chemotherapy (5). Hence, survivin inhibitors could potentially be used in neuroblastoma treatment, especially for high risk patients with poor prognosis (6,7). Anti-BIRC5 antisense based therapies such as gataparsen sodium (LY2181308) and small molecular inhibitors of anti-apoptotic proteins are currently being tested in phase I/II clinical trials (8–10).

**Fig. 1:**
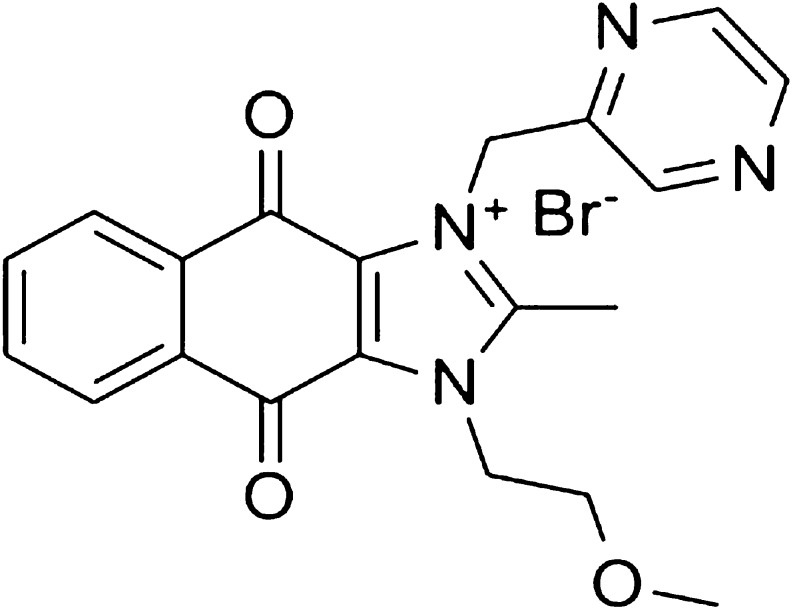
Molecular structure of Sepantronium bromide (YM155).

One of the promising small molecular inhibitors of survivin is sepantronium bromide (YM155) of which the structure is shown in Fig. 1 (11). Recent phase I/II ‘single agent’ clinical trials have shown acceptable toxicity in patients with advanced solid malignancies (10,12). The physicochemical and pharmacological properties of YM155 are listed in Table I. Due to the hydrophilicity and permanent cationic charge on one of the nitrogen atoms of the molecule, YM155 is rapidly eliminated via organic cation transporter (OCT) mediated excretion in bile and urine. Because of this, YM155 has a short plasma half-life of approximately 1–2 h, as determined in pharmacokinetic measurements in mice and rats (13,14). The rapid elimination of YM155 dictates administration as intravenous infusion rather than intravenous bolus injection (12,15).

**Table I:** Characteristics of YM155

Molecular weight	443,3 g/mol (Bromide salt)
LogP	−3.7
pKa	5.3
Solubility in water	At least 0,5 mg/ml
IC_50_	Low nM range for most neuroblastoma cell lines

Nano-encapsulation technologies and application of the resulting so-called nanomedicines in health care have received much attention in recent years, in particular for application in cancer therapy (16,17). Long circulating nanomedicines such as PEGylated liposomes can improve tumor drug delivery by virtue of the enhance permeability and retention (EPR) effect, i.e. the accumulation and retention of nanocarriers in the tumor site due to differences between normal vasculature and tumor blood vessels (18). Thus, the rapid elimination of YM155 and its poor pharmacokinetic properties can potentially be improved by encapsulating the YM155 molecules in a nanocarrier that can prevent renal excretion of the compound. In recent studies, such a PEGylated liposomal dosage form of YM155 has been introduced and studied in rodent cancer models for tumor drug uptake and efficacy (19,20). We now propose a novel immunoliposomal formulation for YM155, which can further improve its delivery to neuroblastoma.

Targeted liposomes (immunoliposomes) have been modified with ligands such as antibodies (Ab) that bind to cell-surface exposed receptors on target cells. Such a binding of the nanomedicine to receptors can be an efficient strategy to facilitate the intracellular delivery of drugs into target cells, which can further enhance their specificity for the targeted cell type (21,22). Disialoganglioside GD2 is an attractive target for an immunoliposome based strategy since GD2 is extensively expressed by neuroblastoma tumor cells (23–26), while it is virtually absent in nonmalignant tissues outside the central nervous system (CNS) (27). GD2-positive tumor cells can be recognized specifically by anti-GD2 antibodies (Ab) or anti-GD2 Ab functionalized nanoparticles (28). Chimeric and humanized anti-GD2 antibodies have been investigated for immunotherapy, tumor vaccination and as targeting ligand for drug delivery purposes (29). Although GD2 is expressed in neurons, the human brain is protected from parenteral anti-GD2 Ab and anti-GD2 decorated nanoparticles by the blood-brain barrier.

In this study, we investigate the formulation of YM155 in GD2-targeted immunoliposomes for the specific delivery of YM155 to neuroblastoma tumor cells. The obtained formulation was evaluated for its in-vitro stability and YM155 release kinetics at different release conditions. The cell specific binding and uptake of the liposomal formulations (i.e. targeted and non-targeted control) were analyzed in cultured NB tumor cells, followed by an in-vitro efficacy evaluation of the YM155 liposomes and free YM155. Finally, a pharmacokinetic pilot study was conducted to investigate the plasma half-life and tumor accumulation of the YM155 immunoliposomes in mice.

## Materials and Methods

### Materials

The lipids 1,2-dipalmitoyl-*sn*-glycero-3-phosphocholine (DPPC), 1,2-distearoyl-*sn*-glycero-3 phosphoethanolamine-N-[maleimide(polyethylene glycol)-2000] (DSPE-PEG_2000_-Mal), 1,2-distearoyl-*sn*-glycero-3-phosphoethanolamine-N-[methoxy(polyethylene glycol)-2000] (DSPE-PEG_2000_) and 1,2-dimyristoyl-*sn*-glycero-3-phosphoethanolamine-N-(lissamine rhodamine-B sulfonyl) (Rho-PE) were purchased from Avanti Polar Lipids (Alabaster AL, USA). Cholesterol (Chol) was obtained from Sigma Aldrich (St. Louis MO, USA). Sepantronium bromide (YM155) was purchased from Bio-connect (Huissen, The Netherlands). Anti-disialoganglioside GD2 (Anti-GD2) antibody was obtained from BD Pharmingen (Alphen aan den Rijn, The Netherlands). N-succinimidyl S-acetylthioacetate (SATA) and Hoechst 33,342 Fluorescent Stain were obtained from Thermo Fischer Scientific (Landsmeer, The Netherlands).4′,6-Diamidino-2-phenylindole (DAPI) was obtained from Roche (The Netherlands). FluorSave mounting agent was obtained from Merck Millipore (San Diego, CA, USA). All chemicals used were analytical grade unless otherwise stated. Cell culture media and supplements were obtained from Gibco laboratories (Gaithersburg, United states). Fetal calf serum (FCS) was obtained from Sigma Aldrich.

### Preparation of Liposomes

Immunoliposomes were prepared from a mixture of DPPC, Cholesterol, DSPE-PEG_2000_ and DSPE-PEG_2000_-Mal in molar ratios of 1.36:1.36:0.14:0.14, based on prior immunoliposomal formulations developed in our department (30). Control liposomes consisted of DPPC, Cholesterol and DSPE-PEG_2000_in molar ratios of 1.36:1.36:0.28. Fluorescently labeled liposomes were prepared by adding rhodamine-PE to the lipid mixture at 0.2 mol% of total lipid (TL). Lipids were dissolved in chloroform: methanol (9:1, *v*/v) and to a final concentration of 90 mM TL. YM155 was co-dissolved with the lipid mixture at a concentration of 0.25 μmol/μmol TL. Organic solvents were removed using a rotary evaporator and subsequent drying under a nitrogen flow. The resulting drug-lipid film was hydrated by adding 3 ml of HBS buffer pH 7.4 (10 mM Hepes containing 135 mM NaCl). Unilamellar liposomes were prepared by multiple extrusion steps over polycarbonate membranes (Nuclepore, Pleasanton, CA, USA) at decreasing pore size range (from 0.4 to 0.1 μm). Next, targeted liposomes (but not control liposomes) were reacted with Sata-anti-GD2 antibody at a final concentration of 30 μg/μmol TL (2 mg/ml antibody) that had been deprotected with hydroxylamine 0.5 M for 45 min prior to its addition to the liposomes. Coupling of S-acetylthioacetyl (SATA) to anti-GD2 (8:1 SATA:Ab mol:mol ratio) was performed as described previously (31). Remaining non-reacted antibody and non-encapsulated YM155 were removed by ultracentrifugation at 60,000 g at 4°C for 30 min and resuspension of liposomes in 3 ml HBS This step was repeated in three times. After the final purification step, liposomes were stored under nitrogen atmosphere at 4°C in the dark for a maximum of 4 weeks prior to usage.

### Characterization of Liposomes

Liposome size and polydispersity was measured using dynamic light scattering (DLS) on an ALV CGS-3 system (Malvern Instruments, Malvern, UK). Zeta-(ζ) potential of liposomes was measured using a Malvern Zetasizer Nano-Z (Malvern Instruments) with universal ZEN 1002 dip cells and DTS (Nano) software. The total lipid concentration was determined according to Rouser (32). YM155 content of the liposomes was determined from 100 μl aliquots which were disrupted by diluting in 300 μl of acetonitrile (ACN). YM155 content was measured with a UPLC Waters Acquity system (Waters Corporation, Milford, MA, USA) equipped with Acquity UPLC ®BEH C18, 1.7 μm column (2.1 × 50 mm) thermostated at 50°C and a UV detector (Waters Corporation, Milford, MA, USA) operated at 252 nm. The gradient mobile phase was operated at a flow rate of 0.5 ml/min and consisted of 5% (*v*/v) ACN in water containing in total 0.1% (v/v) trifluoroacetic acid (solvent A) and 100% ACN containing 0.1% trifluoroacetic acid (solvent B). YM155 encapsulation efficiency (EE %) was calculated from the determined YM155/TL ratio *versus* the initial drug/TL ratio. Conjugation of anti-GD2 to DSPE-PEG_2000_-Malwas confirmed by western blotting using anti-mouse IgG immunodetection. In brief, reduced samples were subjected to SDS-PAGE using 4–12% gradient NuPAGE Novex Bis-Tris mini-gel (Thermo Fischer Scientific). Samples were electrotransferred onto a nitrocellulose membrane via an iBlot Dry Blotting system (Invitrogen). Next, the membrane was blocked with 5% (*w*/*v*) BSA in Tris-buffered saline containing 0.1% Tween-20 (TBS-T) and incubated with goat-anti mouse-IgG secondary antibody conjugated to horseradish peroxidase (HRP) (Thermo Fischer Scientific), diluted 1:1000 in 5% BSA in TBS-T. Proteins were visualized and detected using SuperSignal West Femto Chemiluminescent Substrate (ThermoFischer Scientific) and a Gel Doc imaging system equipped with a XRS camera and Quantity One analysis software (Bio-Rad, Hercules, CA, USA). Coupling of antiGD2 to targeted liposomes was quantified were further characterized by micro-BCA assay (Pierce Biotechnology, Rockford, IL, USA) using mouse IgG (Sigma Aldrich) as calibration standards. Antibody coupling degree (antibody/liposome; ƿ) was calculated using geometric arguments according to Adrian (33) using the following formula: *ƿ* = π/6 × C_Ab_ × (3d_bl_ × R^2^ – 3R × d_bl_^2^ + d_bl_^3^) × M_ab_^−1^× V_Ls_^−1^, in which (C_Ab_) is the measured concentration of coupled antibody (g/mol TL), (M_Ab_) is the molecular mass of the antibody, (R) is the average diameter of liposomes (nm) of the spherical liposomes and (V_Ls_) and (d_bl_) represent the specific lipid volume and lipid bilayer thickness, respectively.

### Storage Retention of YM155 Liposomes

The storage retention of encapsulated YM155 in liposomal formulations in HBS buffer was studied at 4°C for a total duration of 4 weeks. At 0, 5, 10, 20 and 30 days samples were collected in duplicate. Released (non-encapsulated) YM155 was separated from liposomal YM155 by ultrafiltration using Vivaspin centrifugal concentrators with a molecular weight cut-off membrane of 10 kDa (Sartorius AG, Aubagne, France) for 5 min at 4000 g at 4°C. YM155 was determined in both the filtrate (free YM155, released fraction) and the remaining fraction in the upper compartment of the Vivaspin device (total YM155, free and liposomal fraction) which represented at least 70% of the starting volume. The collected samples (both fractions) from each time point were stored in the dark at 4°C for further analysis. Aliquots of 50 μl were taken and diluted in 150 μl ACN, centrifuged at 22,000 g for 10 min at 4°C and subjected to UPLC analysis as described above. Experiments were repeated two times with individually prepared batches of liposomes. Particle size and polydispersity were studied at the beginning and end of the study by DLS as described above.

### In-Vitro Release of YM155 from Liposomes

Release studies were conducted to study the influence of serum proteins and temperature on retention of YM155 in liposomes. Aliquots of anti-GD2 targeted and control liposomes with a final YM155 concentration of 25–30 μM were prepared by diluting the stock solutions prepared for each batch with incubation media consisting of either HBS buffer, 5% bovine serum albumin (BSA) in HBS buffer, or pooled female mouse serum containing *EDTA*-*disodium* (Seralab Laboratories, Haywards Heath, UK) diluted 1:1 with HBS buffer. Samples were incubated at either 37°C or 4°C while gently shaking on a roller bench for a total duration of 24 h. At pre-determined time periods of 6, 12 and 24 h, samples were removed from the incubators and concentrations of released YM155 and total YM155 were determined as described above. Experiments were repeated two times with individually prepared batches of liposomes. YM155 retention in liposomes at each time point was calculated after correction for low levels (<5%) of free YM155 at the beginning of the incubations. The YM155 release data were fitted with zero-order or first order kinetic models, thereby using the following formulas:

$$ {\displaystyle \begin{array}{l}\mathrm{Zero}\hbox{-} \mathrm{order}\ \mathrm{kinetics}:{M}_t/{M}_0=-{k}^{\ast }t\\ {}\mathrm{First}\hbox{-} \mathrm{order}\ \mathrm{kinetics}:{M}_t/{M}_0={e}^{-k\ast t}\end{array}} $$

Where *M*_*0*_ is the amount of encapsulated YM155 at the start of the incubation and *M*_*t*_ is the amount of encapsulated YM155 at each time point; *k* is the release rate constant. The release rate constants (*k)* reported in this work are calculated from first-order kinetics fits to experimental release data. The calculated release rate constants allow a quantitative comparison of the experimental YM155 release profiles of the (liposomal) formulations under the various conditions.

### Culturing of Tumor Cell Lines

Neuroblastoma tumor cell lines KCNR, IMR32 and WiDR *colon adenocarcinoma cells* were obtained from ATCC™. Cells were cultured in Dulbecco’s Modified Eagle’s Medium (DMEM) containing D-glucose (4.5 g/L) and 2 mM glutamate, without pyruvate, and supplemented with 10% fetal calf serum (FCS), 1% MEM Non-Essential Amino Acids (NEAA) and 1% L-Glutamine. Cells from passage 5 to 7 were seeded one day before the experiment in culture flasks (T-75 cm^2^) at 2–4 × 10^6^ cell densities, unless stated differently.

### Assessment of GD2 Expression in Neuroblastoma Cell Lines

Neuroblastoma cell lines were studied for GD2 expression levels by flow cytometry. Cells from each cell line were trypsinized and resuspended at a concentration of approximately 4 × 10^6^ cells/ml in 10 ml of PBS supplemented 6.6% FCS and 20 mM EDTA (flow cytometry buffer). Cells were incubated with either anti-GD2 Ab or mouse isotype control Ab (Thermo Fischer Scientific) as a negative control, at a 1:200 dilution for 45 min at room temperature. Next, cells were washed three times with flow cytometry buffer by centrifugation for 5 min at 300 g and suspended in flow cytometry buffer. After the final washing step, cells were suspended in flow cytometry buffer FACS containing FITC-labeled Goat-anti-Mouse IgG (H + L) secondary antibody (Thermo Fischer Scientific) at a dilution of 1:960, followed by incubation at room temperature in the dark for 20 min. Next, the cells were washed as explained above. Gates were set for GD2-positive and negative fluorescence signals, based on the fluorescence intensity obtained from cells as measured by the AccuriTM C6 Flow Cytometer (BD Biosciences, Erembodegem, Belgium). The analysis was based on both living and dead cells. Generally, 10,000 events were acquired per sample. Data were analyzed with CFlow Plus software (BD Biosciences).

### Binding and Uptake Studies with Rhodamine Labeled Liposomes

The binding of rhodamine labeled GD2-targeted and control liposomes to GD2 positive (i.e. IMR32) and GD2 negative (i.e. WiDR) tumor cells was studied by flow cytometry, using an average of 40 × 10^3^ cells/ml in flow cytometry buffer. Cells were incubated with liposomes for 1 h at 4°C in the dark at different lipid concentrations ranging from 0.1 to 2 mM TL. Cells were washed three times with FACS buffer as described in the previous section after which mean fluorescence intensity (MFI) of the rhodamine signal was determined using the AccuriTM C6 Flow Cytometer. Generally, 10,000 events were acquired per sample. Data were analyzed with CFlow Plus software.

The uptake of rhodamine-labeled liposomes was studied using KCNR neuroblastoma cells by fluorescence microscopy using a Leica TCS-SP confocal laser-scanning microscope (Leica, Heidelberg, Germany). KCNR cells were seeded at a concentration of 40 × 10^4^ cells/ml on FluoroDish™ glass bottom petri-dishes and allowed to adhere overnight. Cells were incubated with rhodamine labeled liposomes at a TL concentration of 1 mM for 4 h at 37°C, followed by washing with cold PBS. Cells were fixed (4% (*v*/v) formaldehyde in PBS; 30 mi at room temperature) and nuclei were stained using Hoechst 33,342 (5 min at room temperature). After washing with cold PBS, the petri-dishes were dried and covered with FluorSave mounting agent and kept at 4°C until confocal microscopy analysis. *Z*-*stack* of optical *sections* (11.5 μm in total thickness) was captured with a 20× objective, using 10 sections with a step size of 1.15 μm. In order to illustrate cell uptake, 2D images were captured from the middle section of the Z-stack (i.e. 5th section).

### In-Vitro Effects of YM155 Loaded Liposomes

Efficacy of YM155 loaded liposomes (targeted and non-targeted control liposomes) was studied on KCNR by alamarBlue viability assay (Thermo Fischer Scientific), according to the supplier’s instruction. KCNR cells were seeded at 20 × 10^3^ cells/well in 96 well Greiner polystyrene plates (Sigma Aldrich) in complete DMEM medium and allowed to adhere overnight. The medium was replaced by cell culture medium supplemented with YM155 or YM155 liposomal formulations at concentrations ranging from 0.8 to 100 nM, after which the cell culture were incubated for 24 h at 37°C. Control experiments included incubations with empty liposomes (i.e. without YM155) at equivalent TL concentrations in the range of 0.08–11 μM TL. After 24 h of incubation, medium was replaced with drug-free medium containing alamarBlue reagent (10 vol%), after which cells were incubated for an extra 3 h at 37°C. Fluorescence was quantified with a microplate reader (Mithras LB940) and used to calculate the relative percentage of living cells normalized against control cells that had not been treated with YM155. IC_50_ values were calculated by curve fitting using non-linear regression in Graph Pad Prism 6.0.

### Pharmacokinetic Pilot Study

Animal experiments were conducted in compliance with the national regulations and have been approved by the local ethical committee for animal experimentation. NMRI nu/nu female mice (25 g, Charles River, Massachusetts, USA) were housed in a temperature-controlled room (approximately 22°C) with 55 to 65% relative humidity, a photoperiod of 12/12 h with free access to water and pelleted rodent food. The mice were challenged with KCNR neuroblastoma (passage 1–2) derived from DAG102776 by serial xenotransplantation in both flanks. When the tumor growth reached the size of approximately 8 × 8 mm, mice were randomly divided in three groups that were injected intravenously with either free YM155 dissolved in HBS (1 mg/kg, *n* = 11), YM155 loaded anti-GD2-immunoliposomes (3 μmol TL/kg diluted in HBS, equivalent to 1 mg/kg YM155, n = 11), or an equivalent dose of YM155 loaded control liposomes (n = 11). At designated time points (i.e. 5 min, 9 min, 15 min, 30 min, 1 h, 4 h, 8 h, 24 h, 48 h and 72 h), blood samples (approximately 0.5 ml per sampling time point) were collected in tubes containing EDTA as anticoagulant and mice were sacrificed. Blood samples and excised tumors were stored at −80°C until analysis of their YM155 concentration by liquid chromatography-tandem mass spectrometry (LC-MS/MS) (34).

### Pharmacokinetic and Statistical Analyses

Pharmacokinetic analysis of the in-vivo data was performed using the Pk Solver 2.0 add-in template for Microsoft Excel, as described previously (35). Pharmacokinetic parameters were determined by non-compartmental analysis (NCA) using linear-logarithmic trapezoidal model fitting, or by compartmental analysis (CA) using 1-compartment model fitting.

Statistical significance was analyzed using two-tailed unpaired Student’s t-test. A *p*-value <0.05 was considered statistically significant.

## Results and Discussion

### Preparation and Characterization of Liposomes

YM155 loaded liposomes were prepared by drug/lipid film hydration and extrusion, and subsequently purified by three cycles of ultracentrifugation and re-dispersion of the pelletized liposomes in HBS. SATA-modified anti-GD2 was conjugated to the maleimidyl anchor of DSPE-PEG_2000_-Mal before the ultracentrifugation procedure, thus avoiding an additional purification process to remove non-conjugated anti-GD2 from the final immunoliposomes. The physicochemical properties of the different types of liposomes are given in Table II. The average liposome size determined by DLS was in the range of 140–170 nm, with a corresponding polydispersity index of about 0.1. The prepared liposomes had a slightly negative zeta-potential of around −10 mV. Attachment of anti-GD2 antibody to the surface of the liposomes did not significantly alter their size or surface zeta-potential as compared to control liposomes, and neither did the inclusion of YM155. The average YM155 encapsulation efficiency was found to be around 14%, which is comparable to encapsulation efficiencies of other hydrophilic drugs, resulting in an average loading content of approximately 0.8 mg/ml (1.8 mM encapsulated drug, corresponding to a drug/lipid ratio of 0.035; see Table II). The reported encapsulation efficiency of 14% is comparable to encapsulation efficiencies by film hydration method of other hydrophilic drugs Quantification of attached anti-GD2 by microBCA showed an antibody coupling efficiency of around 60% for both YM155-loaded and empty liposomes, which corresponds to an average of approximately 33 μg Ab/μmol TL. Assuming an average liposomal size of 140 nm, this results in a calculated 16 to 19 antibody molecules coupled per single liposomal particle (see Table III).

**Table II:** Physicochemical Properties of Liposomal Formulations Loaded with YM155 and/or Containing GD2 Antibody on the Surface

Batch	Size (nm)	PDI	Charge (mV)	TL (μmol/ml)	Lipid yield (%)	Encapsulated YM155 (μmol/ml)	Drug / Lipid ratio^a^	YM155 EE (%)^b^
(Empty) control liposomes	140 ± 1	0.10 ± 0.01	−10.0 ± 5.2	56.3 ± 16.6	62.5 ± 18.4	–	–	–
(Empty) anti-GD2 immunoliposomes	147 ± 14	0.10 ± 0.03	−9.0 ± 1.4	56.6 ± 22.5	62.8 ± 24.9	–	–	–
YM155-loaded control liposomes	161 ± 14	0.05 ± 0.01	−8.1 ± 2.1	52.7 ± 13.3	58.7 ± 11.5	1.85 ± 0.24	0.036 ± 0.010	14.3 ± 4.8
YM155-loaded anti-GD2 immunoliposomes	171 ± 10	0.10 ± 0.03	−9.6 ± 0.5	52.5 ± 14.4	60.2 ± 12.3	1.77 ± 0.29	0.035 ± 0.010	13.9 ± 5.0

Data are presented as mean values of 3–5 preparations ± SD

^a^ Drug/Lipid ratio determined after liposomal disruption. The initial YM155/Lipid ratio prior to liposomal formulation was determined to be 0.25, see paragraph 2.2 in main text

^b^ YM155 encapsulation efficiency is defined as: [liposomal drug/lipid ratio/ [initial drug/lipid ratio]

**Table III:** Efficiency of Anti-GD2 Antibody Coupling to Liposomes

	GD2-YM155 liposomes	GD2 liposomes (empty)
μg Ab/ μmol TL	35.7 ± 9.9	30.9 ± 9.8
Ab coupling efficiency (%)^a^	60.1 ± 5.5	58.7 ± 7.8
Ab / liposome^b^	19 ± 5	16 ± 5

Data are presented as mean values of 3 preparations ± SD

^a^ Antibody (Ab) coupling efficiency (%) = (amount of Ab coupled to liposomes/initial amount of Ab added) × 100%

^b^ Number of Ab molecules coupled per liposome, assuming all recovered Ab is attached covalently to liposomes, and assuming an average liposome size of 140 nm

**Fig. 2:**
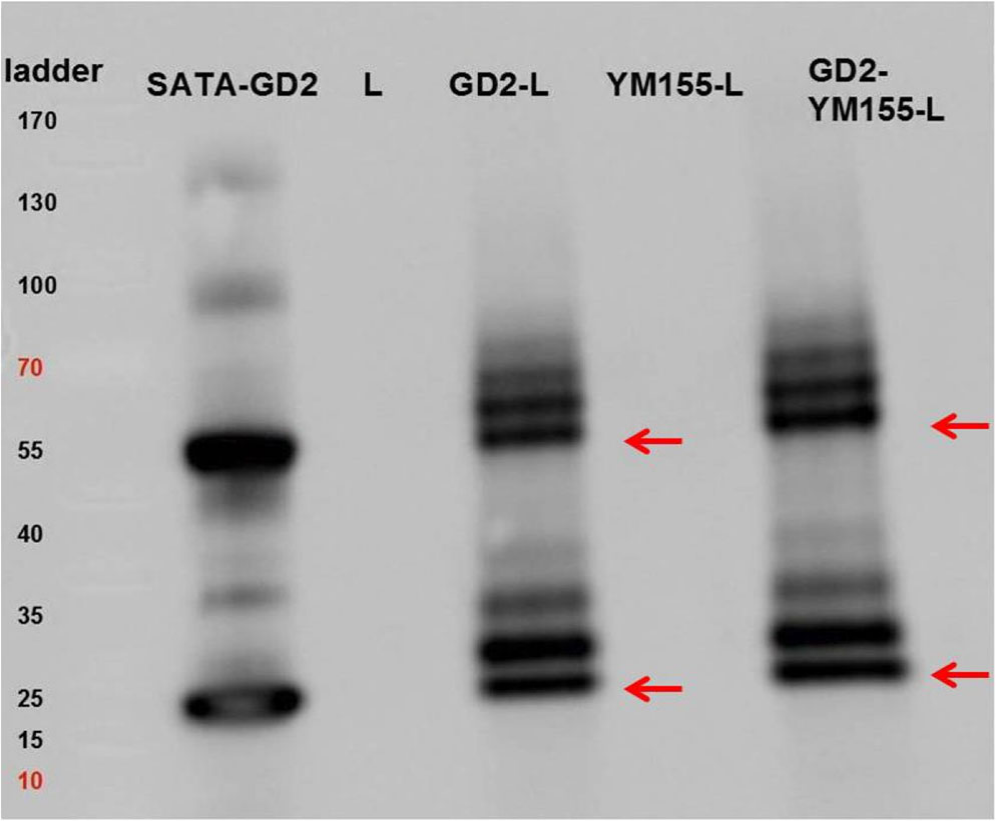
SDS-PAGE (reducing conditions) of: SATA modified anti-GD2 antibodies **(SATA-GD2)**, empty (YM155-free) control liposomes **(L)**, empty (YM155-free) GD2-targeted immunoliposomes **(GD2-L)**, (non-targeted) control YM155 loaded liposomes **(YM155-L)** and GD2-targeted YM155 loaded immunoliposomes **(GD2-YM155-L)** from left to right, respectively. Red arrows indicate the location of bands corresponding to antibody subunits not modified with maleimidyl-PEG-DSPE anchor (see also corresponding bands in SATA-GD2) in liposomal formulations. Protein bands with either 1 or 2 attached PEGylated lipids are clearly visualized at higher MWs. See main text for details.

To confirm that anti-GD2 had indeed been coupled covalently to the liposomal surface, (i.e. by thioether bond resulting from the reaction of SATA-anti-GD2 with the maleimidyl-PEG-DSPE lipids) immunoblotting was performed as described in section 2–3. SATA-modified anti-GD2 Ab (SATA-GD2) migrated as two bands of approximately 25 and 55 kDa, which correspond to the respective light and heavy chains of the antibody (Fig. 2, left). Control (i.e. non-targeted) non-loaded liposomes (L) and control YM155 loaded liposomes (YM155-L) did not display any bands, while both GD2-targeted empty (non-loaded) liposomes (GD2-L) and GD2-targeted YM155 loaded liposomes (GD2-YM155-L) displayed several bands between 25 and 80 kDa corresponding to the molecular weight of anti-GD2 Ab heavy or light chains coupled to one or multiple DSPE-PEG_2000_-Mal anchors (2.9 kDa per unit). Since not all of the anti-GD2 subunits had been modified with SATA and/or conjugated to the maleimidyl-PEG anchor, the immunoliposomal formulations also contained bands that migrated in parallel to the subunits of the parent anti-GD2 protein (i.e. around 25 and 55 kDa).

### Storage Retention and Release of Encapsulated YM155 from Liposomes at Different Conditions

Both liposomal formulations showed good storage stability for at least 3 weeks at 4°C in HBS buffer, as reflected in DLS particle size and polydispersity measurements (Supplemental fig. S3A) and YM155 retention (Supplemental Fig. S3B).

**Fig. 3:**
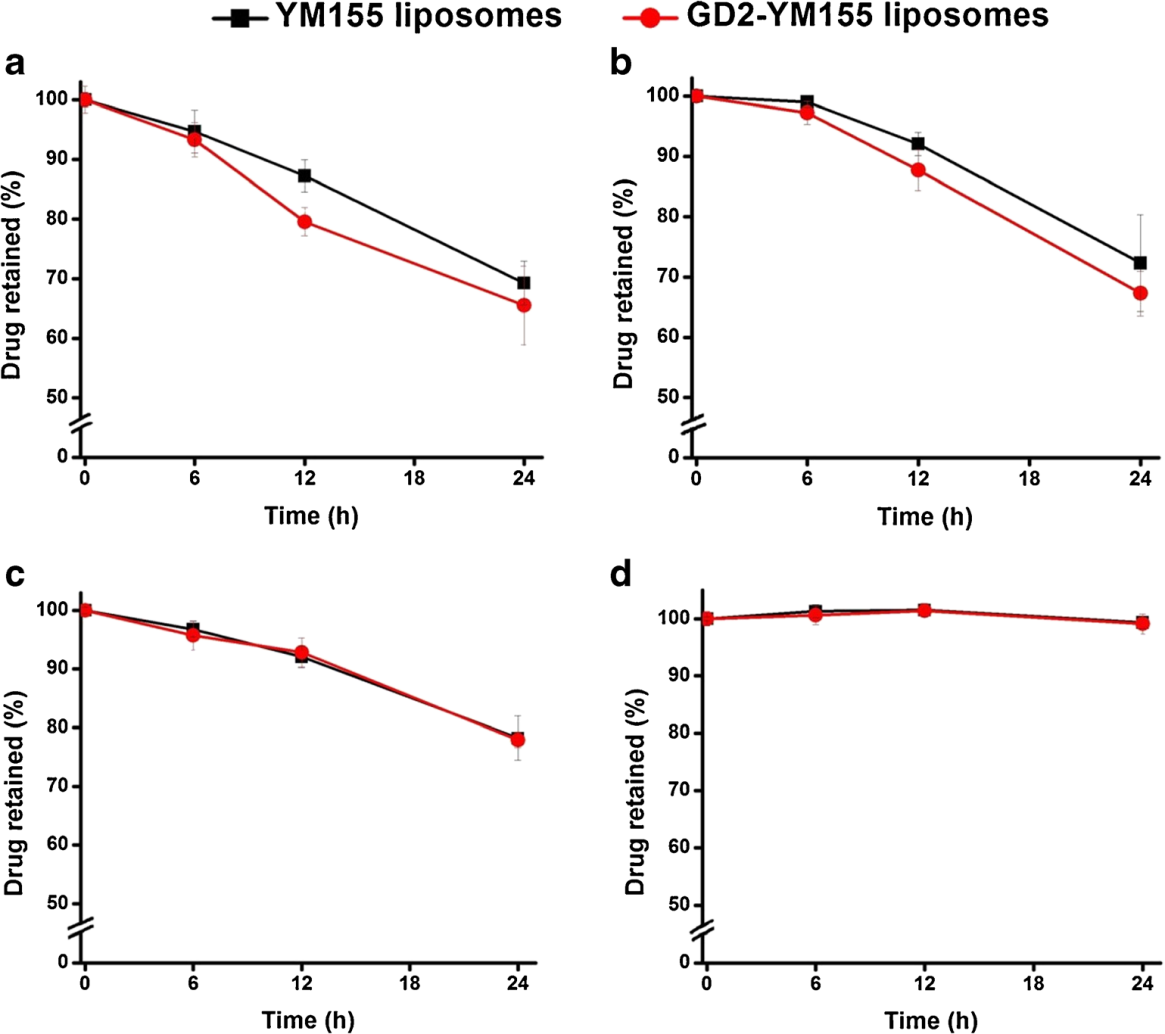
Release experiments were performed at different conditions. (**a**) at 37°C in 5% BSA in HBS buffer, (**b**) at 37°C in 50% serum in HBS buffer, (**c**) at 37°C in only HBS buffer and (**d**) at 4°C in 5% BSA in HBS buffer. Each value represents the mean value (± S.D) of two independent experiments performed in duplicate.

The results of the in-vitro release tests of GD2-targeted immunoliposomes and control liposomes loaded with YM155 are shown in Fig. 3. The conditions of the incubation of panel 3B were chosen to reflect cell culture or in-vivo conditions (50% serum, 37°C), in which effects of either temperature or serum proteins on bilayer stability can be demonstrated. Control and anti-GD2 immunoliposomes released 30.0% ± 11.3 and 34.5% ± 5.2 of the encapsulated YM155, respectively, over the studied 24 h time period. The observed leakage of the liposomes could be largely attributed to temperature-related changes in liposomal stability (Fig. 3c, d, showing release in HBS buffer at 37°C *vs.* 4°C) while the presence of either serum albumin or 50% serum only marginally affected YM155 release (Fig. 3a, b, c showing YM155 release in presence of 5% BSA or 50% serum, *versus* release in HBS buffer without serum proteins). Comparison of first-order release rates (Table IV) confirms a slight increase in the YM155 release rate at 37°C in the presence of serum albumin. The data in Fig. 3 and Table IV also show that there is no significant difference in YM155 release rate between the control liposomes and the anti-GD2 modified immunoliposomes, regardless of the release medium and temperature. Furthermore, it was shown that the YM155 release in the presence of HBS buffer +5% BSA could be halted for at least 24 h if the temperature was dropped to 4°C, as can be seen in Fig. 3d.

**Table IV:** Comparison of the First-Order Rate Constants *k* (h^−1^), Based on Fitting of the in-vitro YM155 Release Profiles Shown in Fig. 3, Showing the Effects of Temperature and Medium Composition on the YM155 Release Rates

Conditions		*k* (h^−1^)^a^	
Temp. (°C)	Medium	Control liposomes	Targeted liposomes
37	HBS +5% BSA	0.018	0.018
37	HBS + 50% serum	0.018	0.021
37	HBS	0.012	0.011
4	HBS +5% BSA ^b^	–	–

^a^ Value of *k* equals the slope of the linear fit (R^2^ > 0.99 in all cases) of the semi-logarithmic plot[(YM155 retained in liposomes) *versus* time] (not shown), extrapolated from the experimental release profiles shown in Fig. 3a-d

^b^ YM155 release from the liposomes in HBS + 5% BSA at 4°C remained unchanged (< 5% for all samples) during a 24 h period, indicating the YM155-containing liposomes are stable in the medium at low temperatures

A possible explanation for the increased YM155 release at higher temperatures is an increase in fluidity of the lipid bilayer at 37°C (36,37). Overall, it can be concluded that YM155 liposomes have moderate stability at body temperature, while neither the presence of proteins (i.e. serum or BSA) in the medium, nor the coupling of anti-GD2 to the liposomal surface appear to greatly influence the stability of the liposomes. The observed release of YM155 from liposomes can be important in the interpretation of other results concerning efficacy and pharmacokinetics, in which free YM155 and encapsulated drug may undergo different uptake and clearance routes. For potential in-vivo applications of YM155-loaded PEGylated liposomes their instability and the related release of the encapsulated drug is not necessarily problematic, as has been reported by Shakushiro and coworkers (19). In their study, YM155-loaded liposomes with various lipid compositions were extensively studied and tested for antitumor activity. Among the investigated formulations 1,2-distearoyl-sn-glycero-3-phosphocholine/ammonium sulfate (DSPC/AS) liposomes were shown to be the most stable formulation, based on the in-vitro YM155 release study. The YM155 release rate from DSPC/AS liposomes reported by Shakushiro and coworkers (19) is comparable to the YM155 release rate of the formulations described here in the present work. However, Shakushiro *et al.* reported that the optimal formulation which demonstrated most potent *in- vivo* antitumor activity was in fact the DSPC/phosphate buffer (BP) liposomes, which showed a relatively higher YM155 in-vitro release rate than the DSPC/AS formulation mentioned earlier. The difference in YM155 release rates between the optimum formulations found previously by Shakushiro *et al.* and the formulations used in this work can be explained by the different nanomedicine targeting approaches in both studies.

Generally, both passively and actively targeting nanomedicine accumulate in the tumor tissue via the enhanced permeability and retention (EPR) phenomenon. When passively targeting nanomedicine accumulates in the target tissue, the encapsulated drug molecules need to be released from their delivery system so that they can be taken up by the target cells. Thus, their release rate needs to be precisely engineered, as described by Shakushiro *et al.* However, for actively targeting nanomedicine direct endosomal uptake of the nanomedicine is facilitated by the presence of (surface) targeting moieties (e.g. anti-GD2 antibody), which is then followed by cytosolic release of the encapsulated drug molecules. Since this study aims to use actively targeting nanomedicine formulations for the delivery of YM155, the more stable liposomal formulations are expected to be most suitable.

### GD2 Expression in Various Neuroblastoma Cell Lines

GD2 expression levels in neuroblastoma tumor cell lines were measured by flow cytometry as explained in section 2–7. KCNR (and IMR32) neuroblastoma showed relatively high levels of GD2 expression (i.e. about 95% and 86%, respectively), while WiDr colon carcinoma cells showed negligible fluorescent signal at the pre-determined gate settings, confirming the lack of GD2 expression on this cell line (data not shown). WiDr cells were further used in flow cytometry experiments as negative control cells.

### Cell Binding and Uptake of Liposomes

**Fig. 4:**
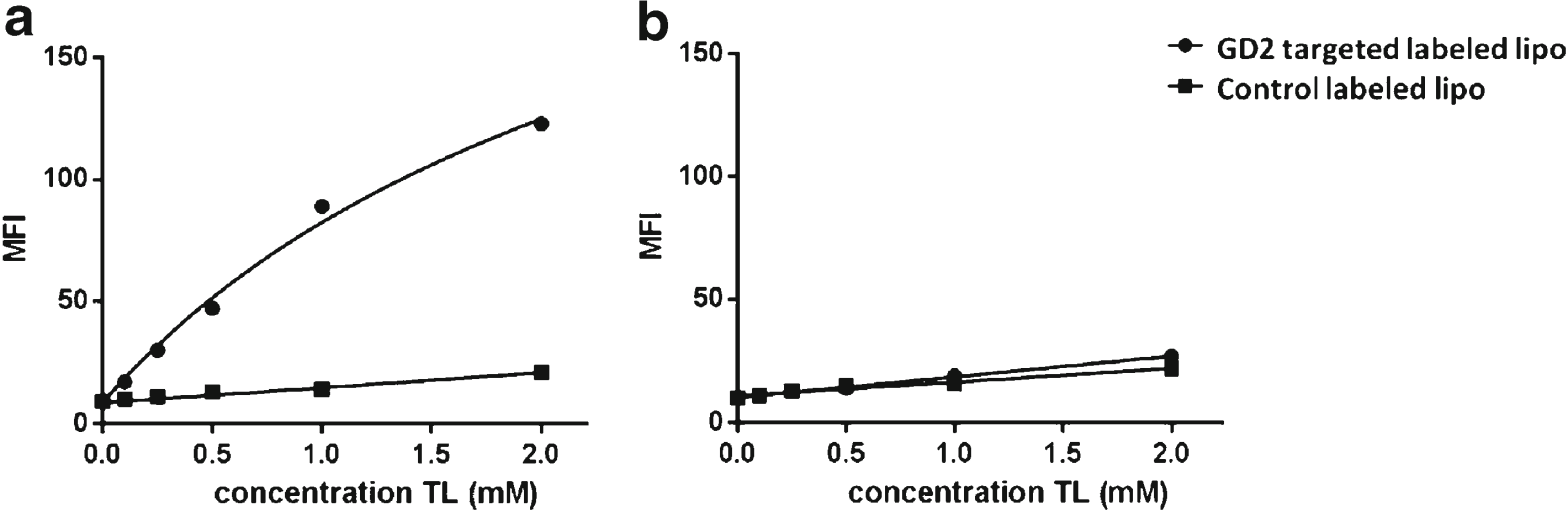
Binding of liposomal formulations to (**a**) IMR32 cells and (**b**) WiDr cells. The data points are aligned (*fitted)* by *non*-*linear regression (using one phase association model).* MFI = Mean Fluorescence Intensity; TL = total lipids.

The ability of GD2-targeted immunoliposomes to recognize GD2 positive cells (IMR32) was demonstrated by incubating rhodamine labeled liposomes with the cells at 4°C, after which cell-associated liposomes were detected by flow cytometry. A four-fold increase in binding of GD2-targeted immunoliposomes to IMR32 cells was observed in comparison to non-targeted control liposomes (Fig. 4a). Moreover, both formulations showed similar low binding to GD2-negative WiDr cells (Fig. 4b), thus confirming the GD2-dependency of liposomal binding.

**Fig. 5:**
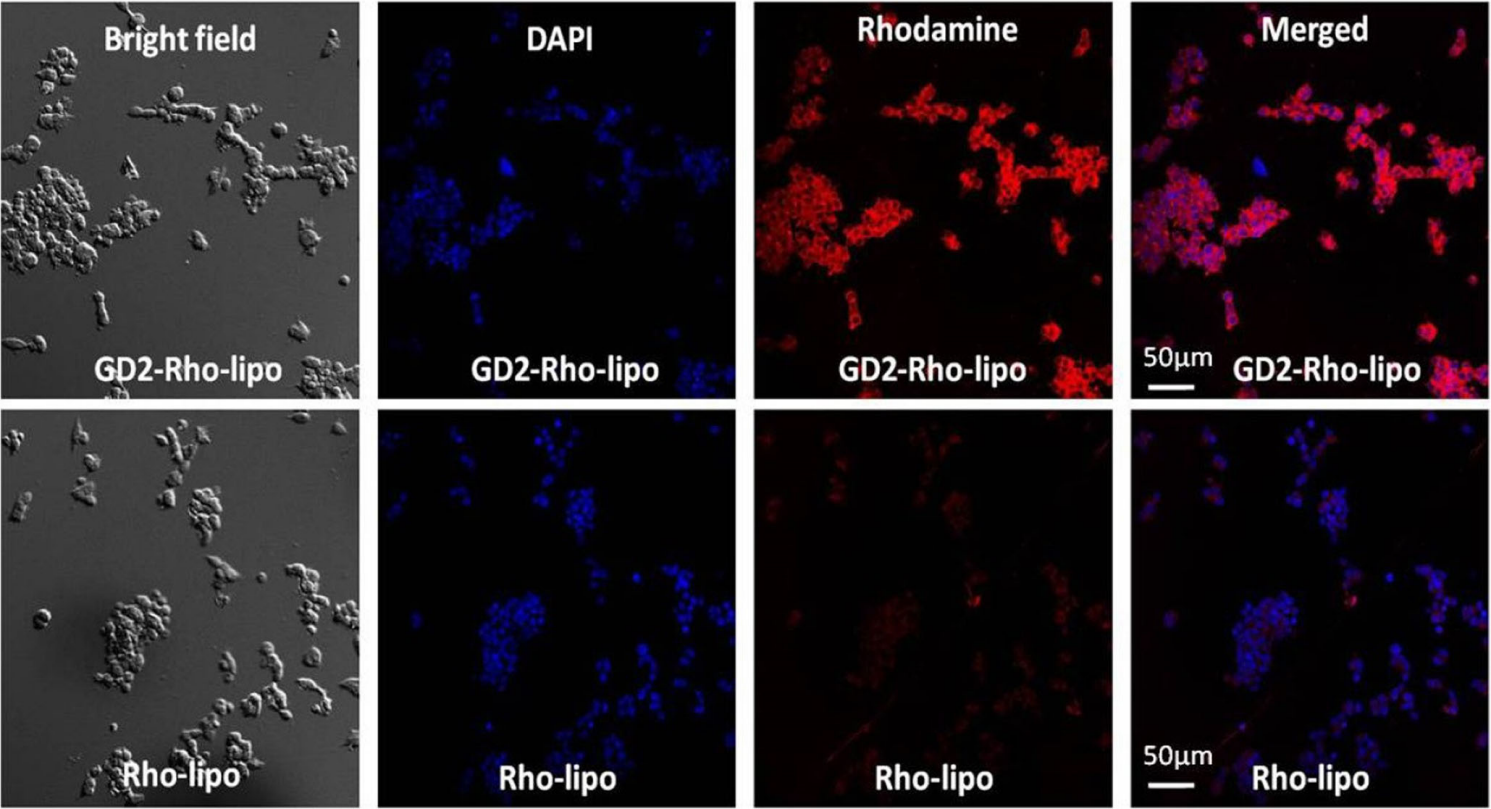
Confocal laser scanning microscope (CLSM) images of KCNR cells incubated with either GD2-targeted labeled liposomes (GD2-Rho-lipo) or with non-targeted control labeled liposomes (Rho-lipo). Blue staining (DAPI) represents cell nuclei. Red staining (rhodamine) represents liposomes. Scale bars in the figures represent 50 μm.

The binding and internalization of GD2-targeted immunoliposomes by KCNR cells was studied by confocal microscopy (Fig. 5). Incubation of cells with liposomal formulation for 4 h at 37°C allowed binding and receptor mediated internalization of immunoliposomes, which was detected by a distinct increase in captured fluorescent intensity at the middle section of z-axis, which was not observed for control liposomes. The obtained results confirm the active (cellular) uptake of GD2-targeted immunoliposomes by KCNR cells.

### In-Vitro Evaluation of GD2 Targeted Loaded Liposomes in Cell Culture

**Fig. 6:**
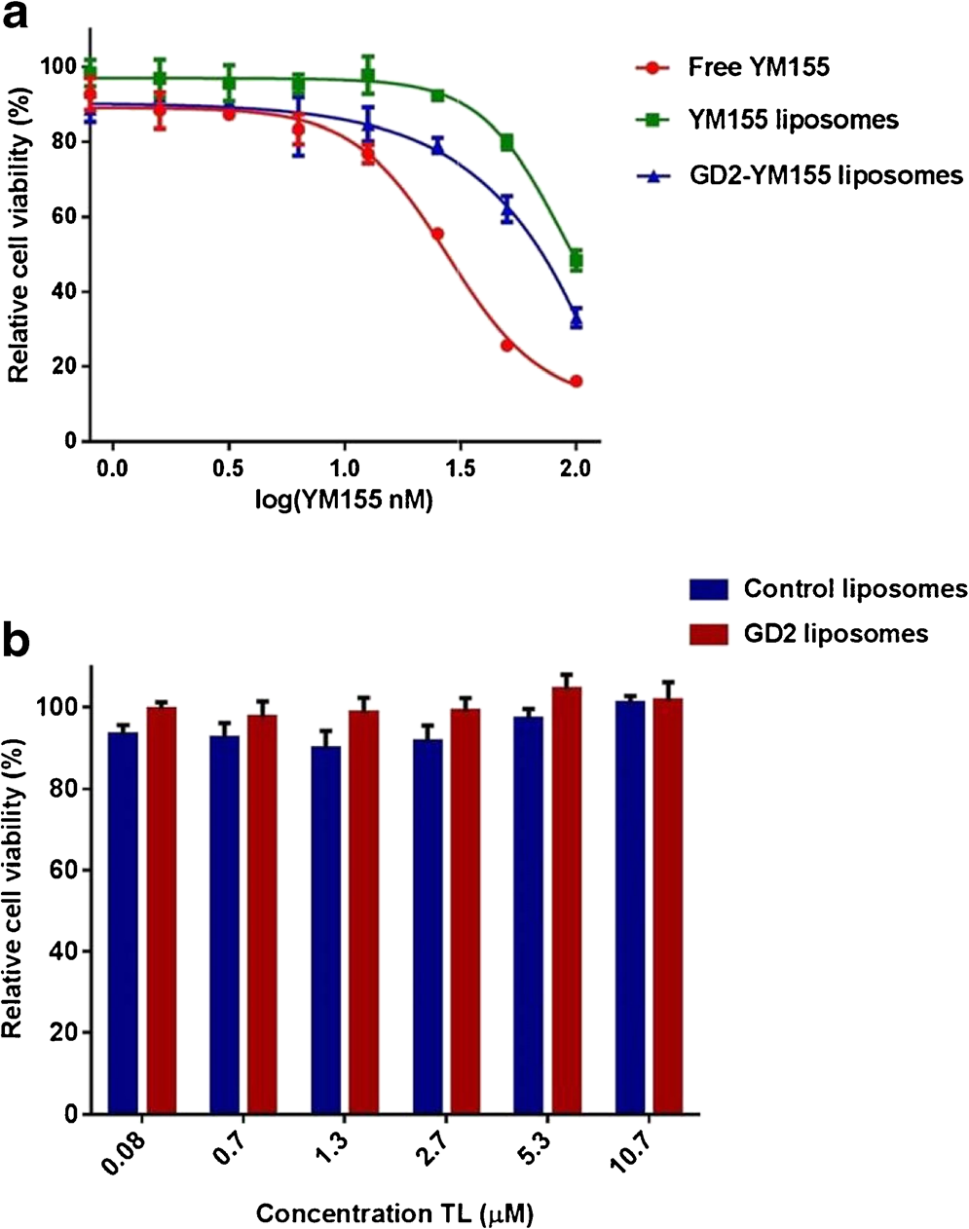
Cell toxicity was determined in neuroblastoma cells (KCNR) via MTS assay after 24 h of total incubation period. (**a**) Semi-logarithmic plot of the cells exposed for 24 h to YM155 loaded liposomal formulations and free YM155. The IC_50_ values were determined with Graph Pad Prism 6.0. (non-linear regression model was used for curve fitting). (**b**) Cells were exposed for 24 h to non-loaded liposomal formulations. Data are plotted as mean values ± SEM (*n* = 3).

The in-vitro efficacy of YM155 loaded liposomes was studied in KCNR cells by incubating the cells for 24 h with the developed formulations. Inhibition of survivin by YM155 will change the balance between cell survival and apoptosis, which can be analyzed via analysis of mRNA or surviving protein expression or apoptosis assays, or indirectly by a cell viability assay (AlamarBlue assay). Dose response curves of cell viability experiments are shown in Fig. 6a and have been analyzed by non-linear curve fitting to determine IC_50_ values of each formulation. IC_50_ values of 27, 60 and 83 nM were calculated for free YM155, anti-GD2 immunoliposomes loaded with YM155, and control liposomes loaded with YM155, respectively. Liposomes not containing YM155 did not induce cell toxicity at the concentrations examined in this work (Fig. 6b). The overall data suggest that treatment of neuroblastoma cells (KCNR) with free YM155 is more effective compared to the treatment of cells with liposomal formulations (targeted or control), although YM155 loaded GD2-targeted immunoliposomes showed an increased (around 20%) efficacy compared to the non-targeted YM155-loaded control liposomes.

**Fig. 7:**
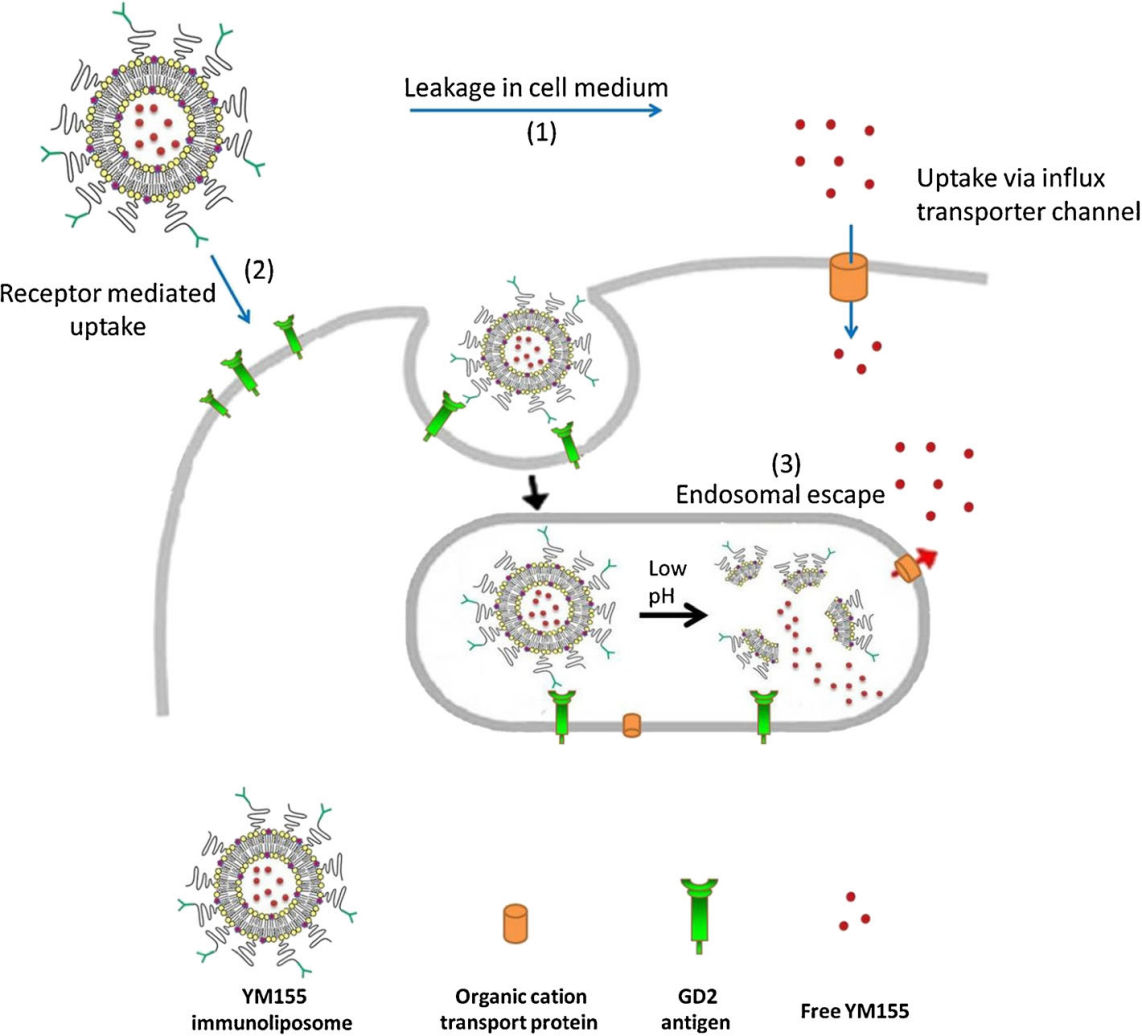
Uptake routes for cytosolic delivery of YM155. Receptor mediated uptake delivers GD2-targeted YM155 liposome into endosomal and eventually lysosomal vesicles. Cytosolic delivery of liberated YM155 is facilitated by passive diffusion (possibly as in complex with an organic anion or via organic cation transporters (OCT) when they are present in the lysosomal membrane). Cytosolic delivery of free YM155 is facilitated via organic cation transport (OCT) channels at the cell membrane. Adapted with permission from ref. (39). Copyright 2006, Springer.

Amphiphilic small molecular inhibitors generally diffuse rapidly through cellular membranes, which enable small molecules to effectively inhibit intracellular molecular targets. However, YM155 is a positively charged small molecule not capable of passive diffusion over lipid membranes. Instead, its intracellular accumulation is actively mediated by cell specific influx transporter channels (38). Apparently, the active uptake of free YM155 is very efficient, outperforming the uptake of immunoliposomes by GD2-positve cancer cells cultured *in vitro*. The major benefit of encapsulating YM155 in liposomes is however in avoiding its rapid renal elimination, which will be presented below. It should thus be noted that part of the effects observed for the liposomal formulations might be due to a fraction of YM155 that is released from the liposomal formulations during the 24 h incubation in protein containing cell culture media at 37°C and which is subsequently transported over the cell membrane similar to free YM155. However, the absence of sample agitation means that YM155 released from the liposomes during the incubation period is likely to be lower than the corresponding YM155 release in the stability tests shown in Fig. 3. More importantly, the difference in efficacy observed between the anti-GD2 immunoliposomes and the non-targeted control liposomes clearly shows that a significant part of the total YM155 is taken up by the cells while remaining encapsulated in the liposomal nanocarrier system. Figure 7 schematically depicts the available uptake routes of YM155 upon its addition to the cells as liposomal formulation or free drug.

The experiments show no toxicity of the anti-GD2 antibodies attached to the surface of liposomes on KCNR cells. However, toxic effects of free anti-GD2 antibodies were previously reported by Gottstein *et al.* on IMR32 cells (40). One cause for these alternate observations regarding anti-GD2 toxicity can be the difference in sensitivity of the neuroblastoma cell types (i.e. IMR32 and KCNR) to anti-GD2 antibody. Another explanation is that Gottstein *et al.* (38) observed toxic effects because in their work the IMR32 cells were exposed to concentrations of anti-GD2 antibody (IC_50_ 1.5 μg/ml) that were much higher than the concentrations of anti-GD2 antibody that the liposomal surfaces were exposed to in this work. The highest concentration of anti-GD2 (coupled to a liposomal surface) that was added to the cell culture media in our experiments was 0.08 μg/ml, which is well below the above mentioned IC_50_ value of 1.5 μg/ml.

### Pharmacokinetic Profiles of Free YM155 and its Liposomal Formulations

**Fig. 8:**
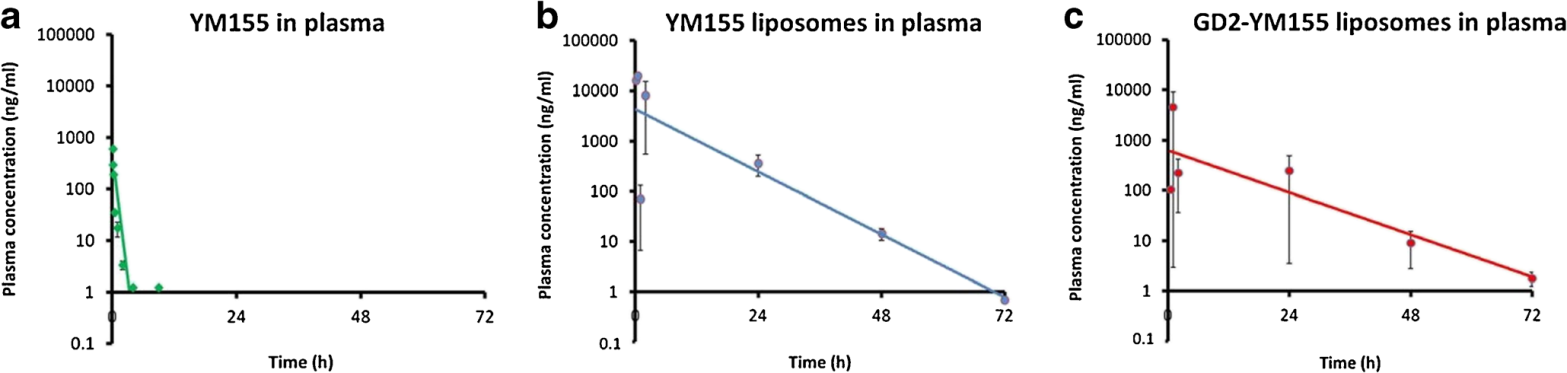
Semi-logarithmic YM155 plasma concentration *vs.* time plots after single i.v. administration in mice of (**a**) free YM155 (**b**) liposomal YM155 in PEGylated liposomes and (**c**) liposomal YM155 in anti-GD2 immunoliposomes. All groups consisted of 11 mice and were treated with a dose corresponding to 1 mg/kg YM155. Each dataset has been fitted using linear regression (solid lines). In (**a**) (free YM155 in plasma) the data point at t = 8 h was excluded to improve the accuracy of the fit. See also Table V and main text.

The distribution kinetics of YM155-loaded liposomes was evaluated in nude mice with subcutaneously implanted neuroblastoma tumors. Upon intravenous injection of either targeted or control liposomes or free YM155, plasma and tumor samples were collected over a time period of 5 min till 3 days and analyzed for YM155 by LC-MS/MS. Figure 8 shows the YM155 plasma concentration *versus* time curves of free YM155 (Fig. 8a), YM155-loaded control liposomes (Fig. 8b), and YM155-loaded anti-GD2 immunoliposomes (Fig. 8c)**.** The calculated pharmacokinetic (PK) parameters for free YM155 and liposomal YM155presented in Table V have been determined by non-compartmental analysis (NCA) using a linear-logarithmic trapezoidal method, in which linear interpolation is used if drug concentrations are increasing or constant (C_i + 1_ ≥ C_i_), while logarithmic interpolation is used if drug concentrations are decreasing (C_i + 1_ < C_i_). The slope of the drug concentration *versus* time curve in the terminal phase (λ_z_) was determined by linear regression on a semi-logarithmic scale, and λ_z_ was then used to derive pharmacokinetic parameters such as drug half-life (t_1/2_) and volume of distribution in the terminal phase (V_z_). PK parameters were also determined by compartmental analysis (CA), in which the YM155 plasma concentration *versus* time curve is fitted using a 1-compartment model. These fitted data are based on 11–13 animals per group, corresponding to 3–5 time points. Combined with the large error margins in some of the data points, the results from the pharmacokinetic analyses listed in Table V should be treated as estimates when comparing them with pharmacokinetic results from other studies. Within this work however, the PK parameters listed in Table V sufficiently show the differences between the three different formulations (i.e. free YM155, Liposomal YM155, and GD2-liposomal YM155). As expected, free YM155 showed a large initial volume of distribution and rapid elimination from the circulation, resulting in plasma levels that were detectable only for about 8 h post administration. In contrast, liposomal formulations of YM155 (targeted and control) displayed much smaller distribution volumes as compared to free YM155, which can be expected for PEGylated nanocarriers that are retained within the circulation initially and that only distribute slowly over the endothelial barrier. Moreover, we observed the expected long-circulating properties of PEGylated liposomes, as both formulations were able to sustain elevated plasma levels of YM155 for at least a 3 day time period (Fig. 8b, c, see also Supplemental Fig. S4 for better comparison during the first hours). The increased longevity of liposomal YM155 in blood plasma can be attributed to the small hydrodynamic size of the liposomes and stealth properties provided by PEG chains, which allow liposomal formulations to escape recognition and subsequent clearance by the mononuclear phagocyte system (MPS) (41,42). As long as the drug is still retained in the liposomal nanocarriers, the normal rapid clearance of free YM155 is prevented.

**Table V:** Pharmacokinetic Evaluation for Total YM155 Levels in Whole Blood after a Single i.v. Administration to Female Mice Bearing Subcutaneous KCNR Tumor Xenografts

	Free YM155 (2 h)^a^ (1 mg/kg)	YM155 liposomes (1 mg/kg)	GD2-YM155 liposomes (1 mg/kg)
t_1/2_ (h)	0.4	5.3	6.8
AUC_0-∞_ (ng/mL*h)	136	71,473	37,787
C_L_ (mL/h)	7337	12	26
V_z_ (mL/kg)	4653	90	258
V_ss_ (mL/kg)	2466	75	98
MRT_0-∞_ (h)	0.34	6.4	3.7

The PK values are calculated by non-compartmental analysis (NCA) using a linear-logarithmic trapezoidal method

*C*_*0*_, concentration at t = 0, extrapolated, t_1/2_ = elimination half-life; *AUC*_*0-∞*_, extrapolated area under the curve from zero to infinity; *C*_*L*_, plasma clearance; *V*_*z*_, volume of distribution during terminal phase, derived from slope (λ_z_) in terminal phase; *V*_*ss*_, volume of distribution at steady state; *MRT*_*0-∞*_, mean residence time

^a^ PK analysis for free YM155 is based on data points until t = 2 h. Inclusion of the t > 2 h data points will result in an unrealistic estimate of the terminal slope λ_z_ (see main text for details)

The difference between free YM155 and liposomal YM155 formulations is also clear when examining the pharmacokinetic parameters derived from the NCA fitting model, as listed in Table V. Clearly, the half-life of free YM155 in blood plasma is shorter (0.4 h) than that of the control liposomes and the GD2-immunoliposomes loaded with YM155 (5.3 and 6.8 h respectively). These half-life values of the liposomes have been derived from the terminal slope (λ_z_) of the semi-logarithmic plots above the detection limit. Of note, plasma concentrations of free YM155 had already dropped below detection level at 8 h and hence only the first few data points (i.e. t ≤ 2 h) have been included to calculate the half-life of the free drug. The half-life of YM155 of 0.4 h is shorter than the previously reported half-life of ~1 h (13). This may relate to differences in experimental setup or the low number of animals in our study. When comparing YM155 loaded GD2-immunoliposomes to control liposomes, a relative large difference in AUC was observed (1.8 fold higher AUC for control liposomes vs immunoliposomes), which was associated with a relative slower elimination of immunoliposomes. As one would expect initial distribution volumes of both types of liposomes to be similar, and maybe faster clearance of immunoliposomes due to the surface modification with antibodies, we cannot fully explain the differences between the two liposomal formulations. A more detailed study on the distribution and elimination of this type of liposomes seems interesting, for instance with labeled liposomes that can be traced immunohistochemically in liver and spleen as these are the main organs responsible for clearance of PEGylated liposomes.

To illustrate the difference in initial and terminal YM155 clearance rates in the different formulations, the pharmacokinetic evaluation was also performed by compartmental analysis (CA), using a 1-compartment model (data not shown). Contrary to NCA, which determines the YM155 half-life from the fitted terminal slope, CA (1-compartment model) fits a first-order exponential decay function to the dataset to determine the PK parameters. The (t_1/2_) values of the YM155 concentration in plasma derived from the NCA and CA methods are listed for comparison in Table VI.

**Table VI:** Comparison of Half-Life Values for YM155 Concentration in Blood Plasma Determined by Non-compartmental (NCA) and Compartmental Analysis (CA)

	NCA t_1/2_ (h)	CA t_1/2_ (h)
Free YM155 (2 h)^a^	0.4	0.2
YM155 Liposomes	5.3	1.9
YM155 GD2-Liposomes	6.8	0.2

Half-life values determined by non-compartmental analysis (NCA) using linear-logarithmic trapezoidal model, and compartmental analysis (CA) using a 1-compartment model. PKSolver CA model fit parameters, correlation coefficient (R^2^) and standard error of weighted residuals (SE): Free YM155 (2 h): R^2^ = 0.81, SE = 153 (ng/ml); YM155 Liposomes: R^2^ = 0.97, SE = 2576 (ng/ml); YM155 GD2-Liposomes: R^2^ = 0.99, SE = 142 (ng/ml).

^a^ PK analyses of free YM155 are based on data points until t = 2 h. See also Table V and main text

It should be noted that the CA used a 1-compartmental model, which means that the fit results are only meaningful for the initial period of rapid decline in YM155 plasma concentration that occurs in the first two hours. Instead, the more gradual decline in the terminal period that can go up to t = 72 h is accurately described by the NCA fit results. The difference is most pronounced for the GD2-liposomes loaded with YM155, for which the rapid decrease observed in YM155 plasma concentration during the first 2 h results in a CA half-life estimate that is even comparable to that of free YM155 (0.23 and 0.20 h, respectively).

### Tumor Accumulation of Free YM155 and its Liposomal Formulations

**Fig. 9:**
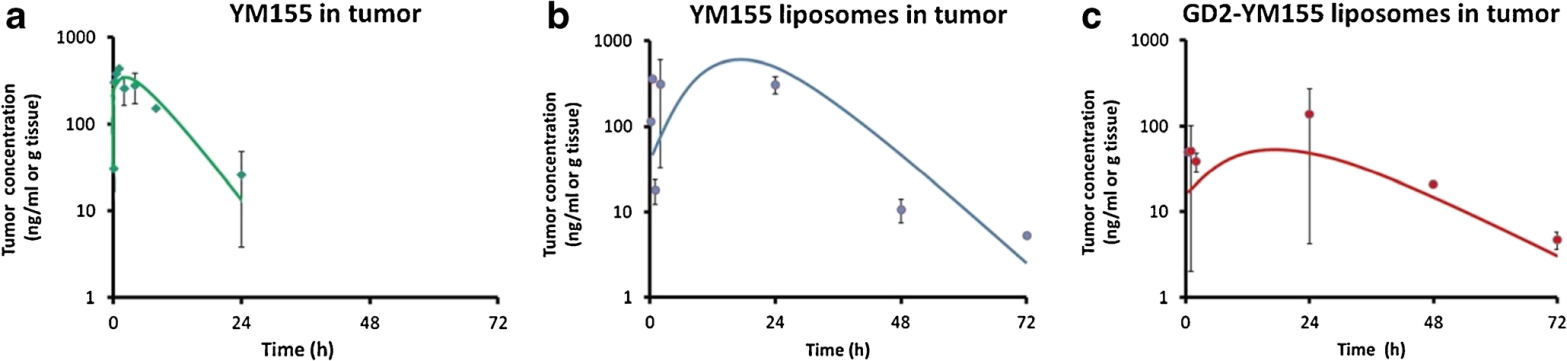
YM155 tumor levels *vs.* time plots after single i.v. administration of (**a**) free YM155 (**b**) YM155 in PEGylated liposomes and (**c**) YM155 in anti-GD2 immunoliposomes. All groups consisted of 11 mice and were treated with a dose corresponding to 1 mg/kg YM155. Each dataset has been fitted using linear regression (solid lines).

As can be observed in Fig. 9, intratumor levels of YM155 could be detected up to 24 h post injection for free YM155, or up to three days post injection for liposomal YM155. It is important to notice that free YM155 was eliminated more slowly from tumor tissue than from the circulation which can be attributed to the cation-transporter mediated uptake of the drug into (tumor) tissue and its subsequent intracellular retention. Tumor accumulation of liposomal YM155 was delayed but sustained as compared to free YM155, which can be attributed to accumulation of nanocarriers via leaky tumor blood vessels, i.e. by EPR, and the prolonged residence of the nanocarriers in the circulation (43,44). Moreover, when comparing anti-GD2 immunoliposomes and non-targeted control liposomes, highest intratumoral levels were observed for control liposomes as compare to targeted liposomes (AUC_0-∞_ values of 13,194 ng/mL*h and 3803 ng/mL*h for control liposomes and anti-GD2 immunoliposomes respectively). The 3.4-fold difference in tumor accumulation cannot be explained fully by the 1.9 fold difference in plasma exposure which should reflect the driving force for tumor uptake by EPR (plasma AUC 71473 ng/mL*h and 37,787 ng/mL*h, for control- and anti-GD2 liposomes respectively). Additionally, one may expect a faster uptake by tumor cells of anti-GD2-liposomes via receptor mediated uptake and intracellular processing of anti-GD2 liposomes, which will liberate YM155 from the liposomal carrier. In contrast, control liposomes will be internalized to a lesser extent in the tumor, and YM155 will remain entrapped inside the liposomal vesicles. Although it was not possible in the context of this pilot study, it would also be very interesting to do an additional study comparing the anti-tumor effects of both liposomal formulations, in order to assess the added value of anti-GD2 immunoliposomes over conventional long-circulating PEG-liposomes for the delivery of YM155.

Liposomal formulations of YM155 have been previously studied, for example in papers by Kawana *et al.* (20) and Shakushiro *et al.* (19). In the study by Kawana and coworkers, the authors developed a liposomal YM155 formulation composed of DSPC, cholesterol and DSPE-PEG_2000_ lipids using phosphate buffer as liposomal ‘inner’ phase. The reported blood circulation half-life (t_1/2_) was 14.6 and 26.5 h for YM155 liposomes at a dosage of 3 and 9 mg/kg, respectively (20). In another study by Shakushiro and coworkers, the authors mainly focused on evaluating the effect of different liposomal formulations on the pharmacokinetics and efficacy. They reported a t_1/2_ of 24 h at 3 mg/kg dose for a liposomal YM155 formulation composed of DPPC, cholesterol and DSPE-PEG_2000_, and a t_1/2_ of 48 h at 3 mg/kg dose for a formulation composed of DSPC, cholesterol and DSPE-PEG_2000_, both containing phosphate buffer as liposomal inner phase (19). We now observe shorter half-lives of 5.3 h and 6.8 h for non-targeted and targeted liposomal YM155 formulations, respectively, which have been determined at relative lower lipid doses, which may have resulted in relative faster clearance of the liposomes.

## Conclusions

The present study reports on the encapsulation of the survivin inhibitor YM155 in immunoliposomes directed to neuroblastoma cancer cells. Surface bound anti-GD2 antibodies render the liposomes neuroblastoma cell specific (i.e. targeted) which has been confirmed by binding and uptake studies with GD2 expressing tumor cells. Both the immunoliposomes and the non-targeted control liposomes showed similar stability under all conditions, excluding detrimental effects of the surface-conjugated antibodies on liposome stability. The liposomal formulations were studied in a pilot pharmacokinetic experiment which demonstrated their long-circulating character and capability to accumulate intra-tumor. When compared to free YM155 after single bolus intravenous (i.v.) injection, prolonged intratumoral levels were obtained. A clear added value of using anti-GD2 immunoliposomes could not be assessed in the conducted pilot study. Further studies are required to evaluate the pharmacological effects (efficacy) of YM155-loaded anti-GD2 immunoliposomes at different YM155 dose ranges which may disclose more details on the potential of the liposomal YM155 formulations for clinical therapeutic applications.

## ACKNOWLEDGMENTS AND DISCLOSURES

This work has been supported by NanoNextNL, a micro and nanotechnology consortium of the government of The Netherlands and 130 partners (project 03D.07). The authors would like to thank Charlene Ogu for her helps with characterization of SATA modified anti-GD2 antibody.

## Electronic supplementary material

ESM 1 (DOCX 442 kb)

## Abbreviations

Ab: Antibodies
ACN: Acetonitrile
AUC: Area under curve
BIRC5: Baculoviral inhibitor of apoptosis repeat-containing 5’
BSA: Bovine serum albumin
CA: Compartmental analysis
Chol: Cholesterol
CLSM: Confocal laser scanning microscope
CNS: Central nervous system
DAPI: 4′,6-Diamidino-2-phenylindole
DLS: Dynamic light scattering
DMEM: Dulbecco’s Modified Eagle’s Medium
DPPC: 1,2-dipalmitoyl-*sn*-glycero-3-phosphocholine
DSPE-PEG_2000_: 1,2-distearoyl-*sn*-glycero-3-phosphoethanolamine-N-[methoxy(polyethylene glycol)-2000]
DSPE-PEG_2000_-Mal: 1,2-distearoyl-*sn*-glycero-3 phosphoethanolamine-N [maleimide(polyethylene glycol)-2000]
EDTA: Ethylenediaminetetraacetic acid
EE: Encapsulation efficiency
EPR: Enhance permeability and retention
FACS: Fluorescence activated cell sorting
FCS: Fetal calf serum
FITC: Fluorescein isothiocyanate
HBS: Hepes buffered saline
HRP: Horseradish peroxidase
IC_50_: Half maximal inhibitory concentration
IgG: Immunoglobulin G
LC-MS/MS: Liquid chromatography-tandem mass spectrometry
MFI: Mean fluorescence intensity
MPS: Mononuclear phagocyte system
NB: Neuroblastoma
NCA: Non-compartmental analysis
NEAA: Non-essential amino acids
OCT: Organic cation transporter
PEG: Polyethyleneglycol
PBS: Phosphate buffered saline
PK: Pharmacokinetic
Rho-PE: 1,2-dimyristoyl-*sn*-glycero-3-phosphoethanolamine-N-(lissamine rhodamine-B sulfonyl)
SATA: N-succinimidyl S-acetylthioacetate
SDS-PAGE: Sodium dodecyl sulphate polyacrylamide gel electrophoresis
t_1/2_: Half life
TBS-T: Tris-buffered saline containing 0.1% Tween-20
TL: Total lipid
UPLC: Ultra performance liquid chromatography
UV: Ultraviolet
