# Supplementary material for: Anti-GD2 Immunoliposomes for Targeted Delivery of the Survivin Inhibitor Sepantronium Bromide (YM155) to Neuroblastoma Tumor Cells
Source: Pharm Res. 2018 Mar 7;35(4):85. doi: 10.1007/s11095-018-2373-x (PMC5842274; doi:10.1007/s11095-018-2373-x)
Supplement: Supplementary file 1 — (DOCX 442 kb) [file 11095_2018_2373_MOESM1_ESM.docx]

**Anti-GD2 immunoliposomes for targeted delivery of the survivin inhibitor Sepantronium bromide (YM155) to neuroblastoma tumor cells**

Shima Gholizadeh^1^,Emmy M. Dolman^2,3^, Rebecca Wieriks^1^, Rolf W. Sparidans^4^,
Wim E. Hennink^1^, Robbert J. Kok^1^

1. Department of Pharmaceutics, Utrecht Institute for Pharmaceutical Sciences, Utrecht University, Utrecht, the Netherlands
2. Department of Oncogenomics, Academic Medical Center, University of Amsterdam, Amsterdam, the Netherlands
3. Princess Maxima Center for Pediatric Oncology, Utrecht, the Netherlands
4. Department of Chemical Biology and Drug Discovery, Utrecht Institute for Pharmaceutical Sciences, Utrecht University, Utrecht, the Netherlands

**Supplementary information**

**Supplementary materials and methods**

**Determining the functionality of SATA-modified anti-GD2 antibody**

The functionality of SATA-modified anti-GD2 antibody (Ab) was assessed by FACS analysis on KCNR cells. For this study, cells at concentration of 4x10^6^ cells/ml were incubated with native and SATA modified anti-GD2 Ab at 1:200 dilution in FACS buffer. Cells were incubated for 45 minutes at room temperature followed by 3 washing steps with FACS buffer as explained in section 2-8. Next, cells were incubated for 20 minutes at room temperature with FITC labeled goat anti-mouse Ab at 1:960 dilution, followed by washing steps. At the final step, cells were resuspended in FACS-buffer and the fluorescence intensity was measured by AccuriTM C6 Flow Cytometer (BD Biosciences, Erembodegem, Belgium). The analysis was based on both living and dead cells. Generally, 10,000 events were acquired per sample. Data were analyzed with CFlow Plus software.

**Supplementary data**

**
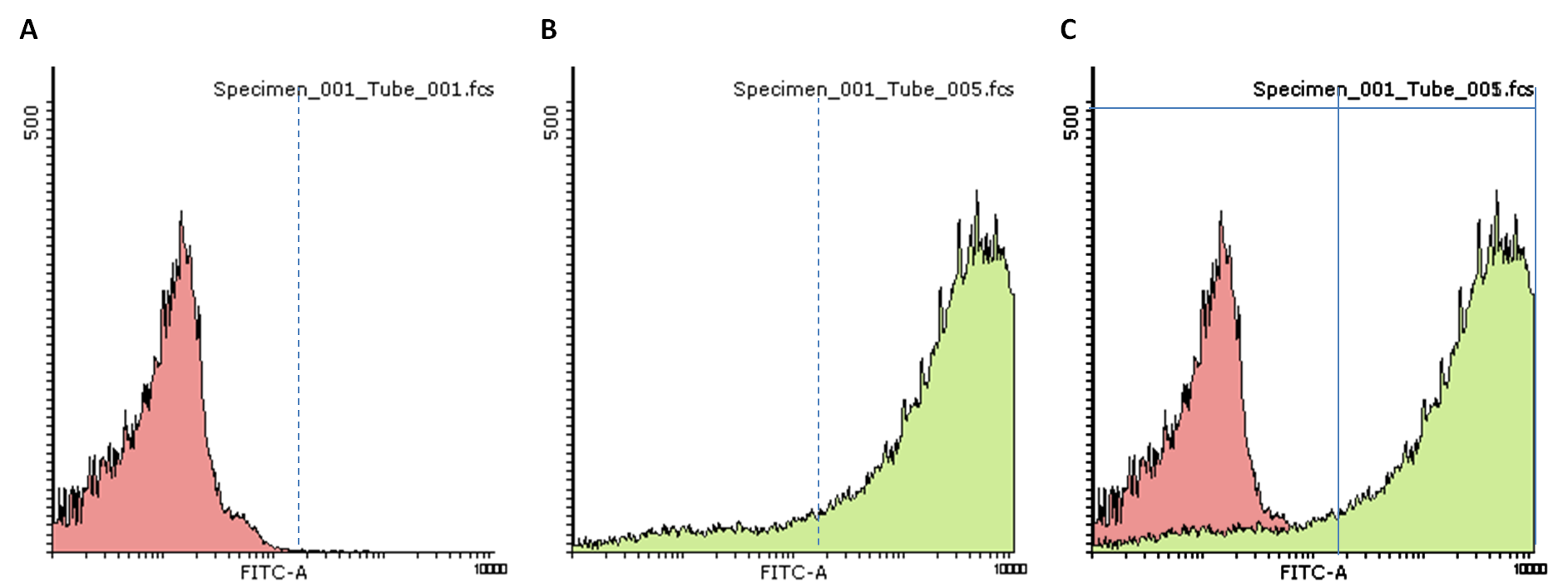
**

**Fig. S1.** Example of flow-cytometry analysis to evaluate GD2 expression on IMR32 cells. **(A)** The staining with control isotype antibody is shown as the solid red profile. **(B)** The staining with the anti-GD2 antibody is shown as solid green profile. **(C)** An overlay of histograms. The percentage of positive expression is based on the area under the curve for the green histogram, on the right side of the blue lined set border.


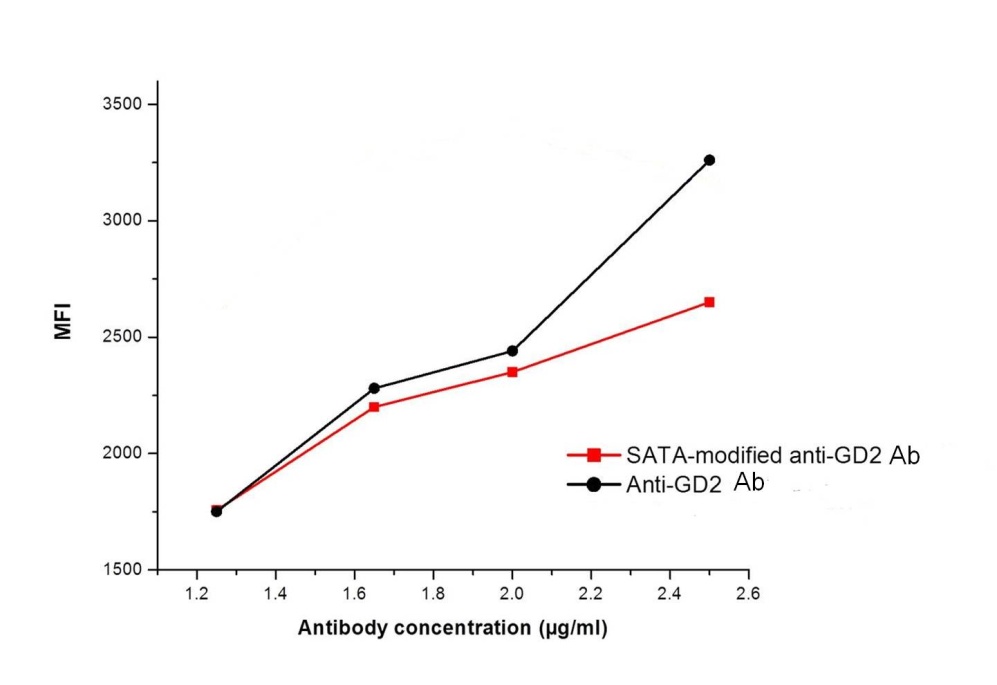


**Fig. S2.** Modification of the anti-GD2 antibody with SATA only had a moderate influence the binding capacity of the antibody to GD2-positive cells.


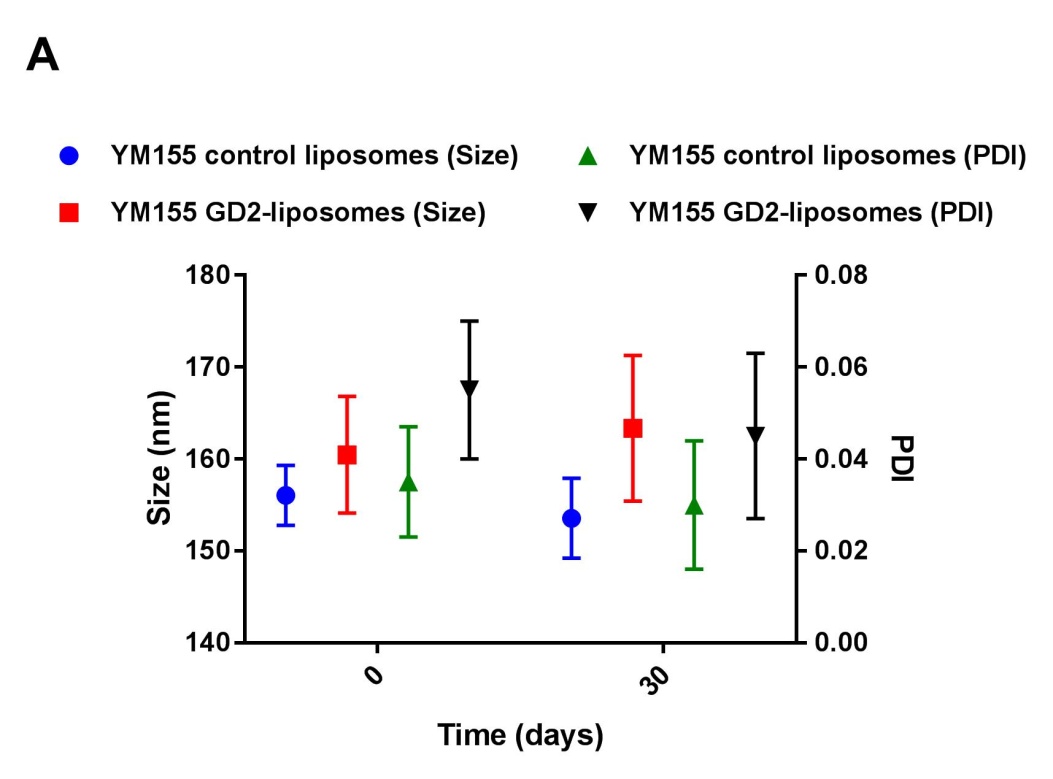


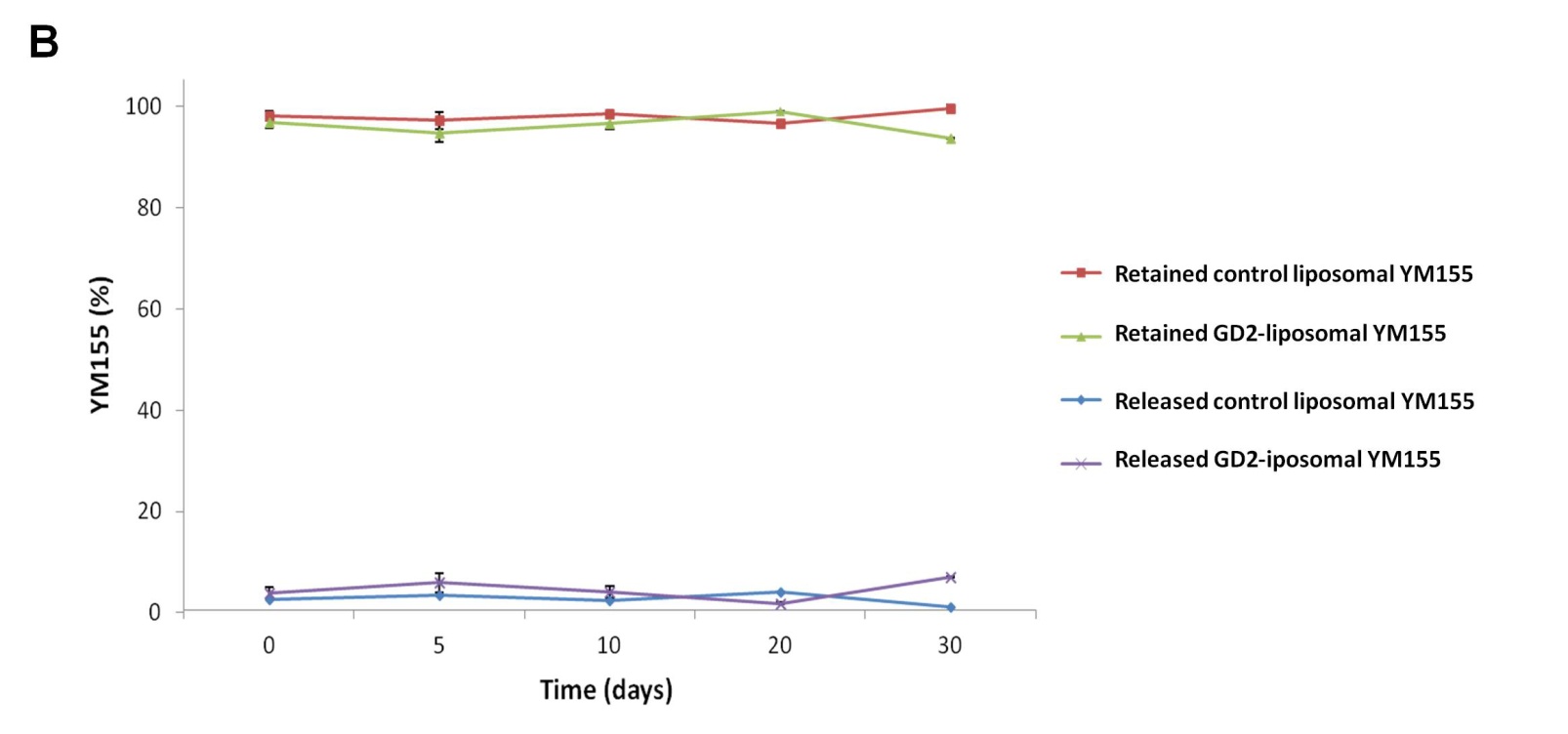


**Fig. S3.** Results of the stability study on liposomes at 4 °C in HBS buffer**. (A)** Measured particle size and polydispersity (PDI) at time 0 and at the end of the study (day 30). **(B)** Measured retained and released YM155 from liposomes during the storage period. Each value represents the mean (± SD) of two independent experiments.


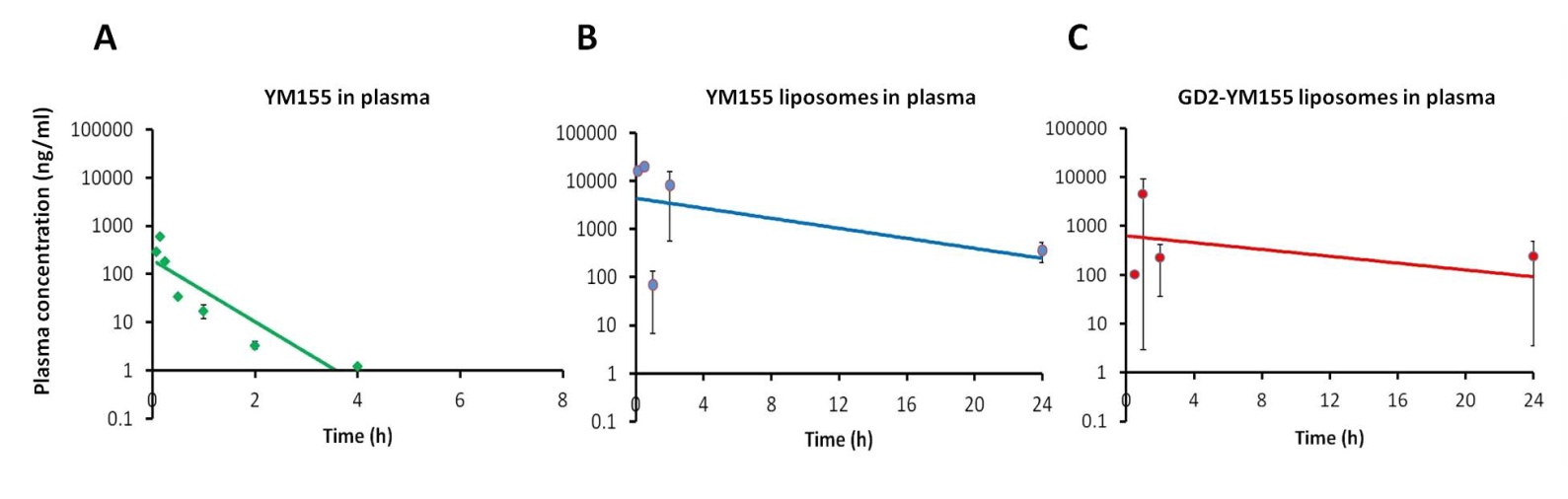


**Fig. S4.** Semi-logarithmic YM155 plasma concentration vs. time plots, during the first 24 h following single i.v. administration in mice of **A:** free YM155 (1 mg/kg) **B:** YM155 loaded in control liposomes and **C:** YM155 loaded in anti-GD2 immunoliposomes. All treatments corresponded to YM155 (1 mg/kg) (n=11 per group).
